# “What Did I Do to Be so Black and Blue?”^1^

**DOI:** 10.3201/eid1306.000000

**Published:** 2007-06

**Authors:** Polyxeni Potter

**Affiliations:** * Centers for Disease Control and Prevention, Atlanta, Georgia, USA

**Keywords:** Jacob Lawrence, marionettes, art and science connection, social realism, dynamic cubism, American art, art and emerging diseases, humanities and science, art commentary, about the cover

**Figure:**
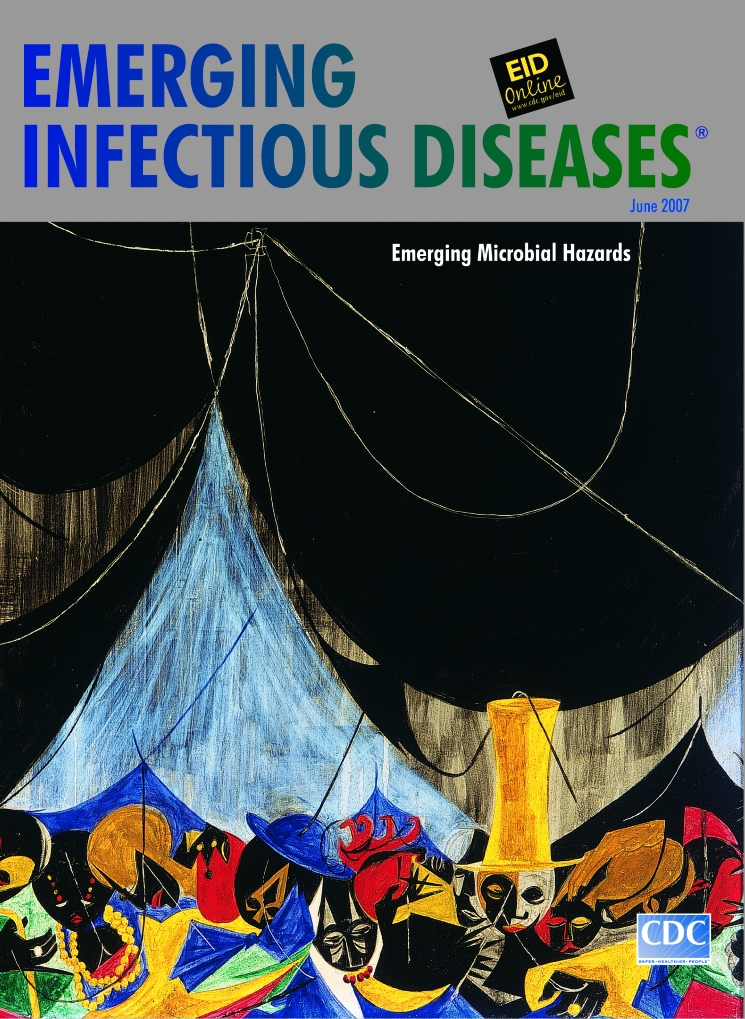
**Jacob Lawrence (1917–2000). Marionettes (1952).** Tempera on panel (46.4 cm x 62.2 cm). High Museum of Art, Atlanta, Georgia, USA. Purchased with funds from the National Endowment for the Arts and Edith G. and Philip A. Rhodes, 1980.224.

—Louis Armstrong

“Our homes were very decorative, full of…pattern…color….The people used this as a means of brightening their life,” said Jacob Lawrence, attributing his love of vibrant color design to his youth in Harlem (*1*). When asked if anyone in his family was artistically inclined, he would say no, “It’s only in retrospect that I realized I was surrounded by art. You’d walk Seventh Avenue and look in the windows and you’d see all these colors in the depths of the Depression, all these colors!” (*1*). “Most of my work depicts events from the many Harlems that exist throughout the United States. This is my genre. My surroundings. The people I know…the happiness, tragedies, and the sorrows of mankind…” (*2*).

Lawrence was born in Atlantic City, New Jersey, “But I know nothing about it,” he always said, because his family soon moved, to Pennsylvania (*3*). He moved again in his early teens, with his mother, after his parents separated. “And we came to New York and of course this was a completely new visual experience” (*3*). Lawrence showed artistic talent at an early age. “I liked design…. I used to do things like rugs by seeing the pattern…in very bright primary and secondary colors…and papier-mâché masks…not for play or anything…I just liked to make them….My first exposure to art which I didn’t realize was even art at the time was at an after-school settlement house….The Utopia Children’s Settlement House” (*3*).

“I never saw an art gallery until I was about eighteen years of age…. And going to the settlement house I was exposed to arts and crafts; soap carving, leather work, woodwork and painting…” (*3*). In the early 1930s, Depression relief programs sprang up all over the United States. Lawrence met Augusta Savage, already a well-known sculptor, at a center across the street from where he lived. He met writers Alain Locke, Richard Wright, and Ralph Ellison and worked with many prominent artists of the day, Norman Lewis, Charles Alston, Romare Bearden, Henry Bannarn.

Encouraged by Augusta Savage, he participated in the Works Progress Administration’s Federal Arts Project, a program founded in 1935 to create jobs in the arts, “…[I]t was like a very informal schooling. You were able to ask questions of people who had more experience …about technical things in painting” (*3*). For inspiration, he visited the 135th Street Branch of the New York Public Library and walked 60 blocks to the Metropolitan Museum of Art.

Lack of academic training did not thwart Lawrence’s artistic development. Rather, his individual style, borne of his personal view of the world and nourished by the community around him, flourished early and well. His flat patterns and colors and bold narrative scenes showed the influence of Mexican painters José Clemente Orozco, David Alfaro Siqueiros, and Diego Rivera, and of Polish artist Käthe Kollwitz, all of whom espoused a social realist philosophy.

At age 21, Lawrence attracted attention with his series of 41 paintings on Toussaint Louverture (c.1743–1803), a hero of the Haitian Revolution. He read voraciously and researched his topics thoroughly. He painted a story over a series of panels planned and executed as a cluster. Each scene was outlined in pencil, each color applied to all panels simultaneously to ensure consistent tonal quality across the series (*4*).

“The Human subject is the most important thing. My work is abstract in the sense of having been designed and composed, but it is not abstract in the sense of not having human content…I want to communicate. I want the idea to strike right away” (*2*). Lawrence freed his figures of detail, exacting the essence, which he punctuated with saturated color recalling Henri Matisse, and he repeated patterns and breaks, creating a rhythmic quality reminiscent of jazz syncopations. In gouache and tempera, he recreated the “hard, bright, and brittle” feel of Harlem during the Great Depression (*2*).

The work that brought Lawrence national recognition was The Migration of the Negro, a series of 60 panels recounting the mass movement of African Americans from the rural South to northern urban centers. The series was featured in Fortune magazine in 1941. He continued to paint for decades, at times doubting the strength of his style within modernism and questioning the influence of popularity on his work. He taught at the Pratt Institute, the New School for Social Research in New York, the Skowhegan School of Painting and Sculpture in Maine, and the University of Washington in Seattle. When he died at 82, Lawrence the chronicler of major cultural events of the 19th and 20th centuries had created an American aesthetic, and his expressive style, crafted in Harlem workshops, had made a lasting impression.

In Marionettes, on this month’s cover, Lawrence revisited a topic addressed in The Dancing Doll (1947), an earlier work he had described as “mostly autobiographical” (*5*).

Marionettes, controlled with strings by a puppeteer from above, predate live theater. They were found in the tombs of ancient Egypt and the works of Archimedes and Plato.

Their inherent inability to stand alone makes marionettes an irresistible artistic and literary metaphor. In Invisible Man (1947), his epic of self-discovery, Ralph Ellison describes his encounter with a marionette, “I’d seen nothing like it before. A grinning doll of orange-and-black tissue paper with thin flat cardboard disks forming its head and feet and which some mysterious mechanism was causing to move up and down in a loose-jointed, shoulder-shaking, infuriatingly sensuous motion, a dance that was completely detached from the black, mask-like face” (*6*).

Viewed en masse at the bottom of the painting, Lawrence’s marionettes seem dwarfed under the dark backdrop and drooping tents, the inevitable strings a reminder of their attachment to a set. In what seems a makeshift theater, they await the next move. To paint the lifeless dolls, the artist was prompted by social ills, which strip people of control over their lives, causing them to withdraw because, as Ellison put it, “…ain’t nothing I can do but let whatever is gonna happen, happen” (*6*).

As with all his work, Lawrence touched a universal nerve: human vulnerability against forces beyond one’s control. “I'm so forlorn, Life's just a thorn/My heart is torn/Why was I born?” lamented Louis Armstrong (1901–1971), speaking for Lawrence and for all of us. Faced with overwhelming social injustice or with recurring insults of a more biologic nature—microbial resistance to drugs, mutating viruses, emerging prions, migrating hazards—we may at times seem little more than hapless marionettes, caught in a degrading tangle at the foot of a large set.
